# Regional Disparities and Factors Associated With Poverty Among Women of Reproductive Age in Somalia: Evidence From National Survey Data

**DOI:** 10.1002/puh2.70435

**Published:** 2026-11-01

**Authors:** Mohamed Mustaf Ahmed, Mohamed Abdirahim Omar, Yahye Sheikh Abdulle Hassan, Abdirasak Sharif Ali

**Affiliations:** ^1^ Department of Data Science SIMAD University Mogadishu Somalia; ^2^ Center for Health Research and Innovation Somali National University Mogadishu Somalia; ^3^ Faculty of Health Science Salaam University Mogadishu Somalia; ^4^ Faculty of Medicine and Health Sciences Jamhuriya University of Science and Technology Mogadishu Somalia; ^5^ Department of Microbiology and Laboratory Sciences Faculty of Medicine and Health Sciences SIMAD University Mogadishu Somalia; ^6^ Somali Research Sharks Network Mogadishu Somalia

**Keywords:** Demographic and Health Survey, health equity, poverty, regional disparities, Somalia, women of reproductive age

## Abstract

**Background:**

Poverty disproportionately affects women through labor inequities, unpaid care burdens, and limited resource control. In Somalia's fragile context, understanding subnational poverty disparities among reproductive‐age women is essential for targeted health interventions.

**Methods:**

A cross‐sectional analysis was conducted using data from the 2020 Somalia Demographic and Health Survey (SDHS). The analytical sample included 13,123 birth records from women aged 15–49 years (unweighted *n* = 13,123; weighted *N* ≈ 12,774) with complete information on all variables included in the analysis. Poverty status was defined as poor versus nonpoor, using the DHS household wealth index (dichotomizing the poorest and poorer quintiles vs. middle, richer, and richest quintiles). Survey‐weighted descriptive analyses, bivariate associations using design‐based Rao−Scott *F*‐tests, and multivariable survey logistic regression were performed in Stata version 18.0 to estimate the crude and adjusted odds ratios (aORs) with 95% confidence intervals (CIs) accounting for clustering, stratification, and sampling weights.

**Results:**

The overall prevalence was 48.5% (95% CI: 42.4%–54.6%), ranging from 28.2% in Bay to 73.8% in Gedo. Primary schooling (aOR = 0.31, 95% CI: 0.23–0.44), secondary schooling (aOR = 0.08, 95% CI: 0.02–0.36), and higher education (aOR = 0.09, 95% CI: 0.01–0.92) were associated with lower odds of poverty. No media exposure (aOR = 4.47, 95% CI: 2.92–6.83), having an unemployed husband (aOR = 4.18, 95% CI: 3.28–5.31), home delivery (aOR = 3.90, 95% CI: 2.94–5.18), and barriers (aOR = 2.36, 95% CI: 1.84–3.02) were associated with higher odds of poverty. Compared with Awdal, the odds of poverty were lower in Bay, Sanaag, Sool, Bakool, Lower Juba, and Banadir.

**Conclusion:**

Nearly half of women in the analytical sample were classified as poor on a relative wealth measure, with marked regional disparities and strong associations with education, information access, household dynamics, and maternal health use. Interventions could prioritize girls’ education, information access, employment, and maternal healthcare in high‐poverty areas.

**Clinical Trial Number:**

Not applicable.

## Graphical abstract

Nearly half of Somali women of reproductive age (48.5%) lived in the poorest two‐fifths of households, ranging from 28.2% in Bay to 73.8% in Gedo. Education was linked to lower odds of poverty, whereas no media exposure, husband's unemployment, home delivery, and healthcare barriers were linked to higher odds.

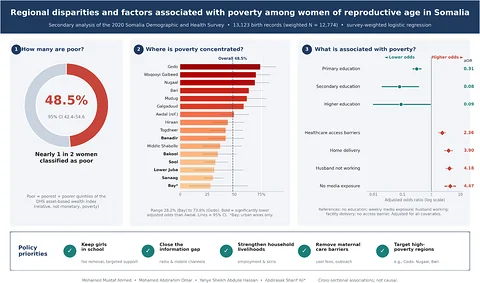

## 1 Introduction

Poverty is increasingly recognized as a multidimensional phenomenon that extends beyond mere income insufficiency to encompass deprivation in health, education, and social agency [1, 2]. This condition is not gender‐neutral; women experience poverty more acutely because of structural inequalities in the labor market, heavy domestic burdens, and limited control over household resources [1, 3]. For women of reproductive age (15–49 years), poverty is a significant barrier to exercising reproductive rights and maintaining health, creating a “poverty trap” that affects both the individual and her offspring [4, 5]. In fragile contexts like Somalia, where natural disasters, conflict, and food insecurity are prevalent, these vulnerabilities are magnified, leading to some of the highest burdens of nutritional deficiency among women of reproductive age worldwide [6].

The relationship between women's health and economic status is reciprocal. Improvements in reproductive health, such as access to family planning and maternal care, have been shown to facilitate women's economic empowerment by enabling higher educational attainment and increased labor force participation [4, 7]. Conversely, socioeconomic adversity, characterized by low education, unemployment, and lack of social safety nets, is a primary driver of poor health outcomes [8]. Evidence from other developing regions indicates that women in lower‐income households are significantly more likely to suffer from chronic energy deficiency, undernutrition, and anemia than those in higher‐income brackets [9, 10]. These health disparities are often mediated by the “motherhood penalty,” where childbearing leads to career interruptions and skill depreciation, further entrenching economic disadvantage [7].

Regional disparities play a critical role in poverty and health outcomes distribution. Research in sub‐Saharan Africa has highlighted significant variations in health insurance coverage and service utilization between urban and rural settings [11]. Rural women often face a “double burden” of agricultural and domestic labor while residing in areas with deficient healthcare infrastructure and limited access to clean water [8, 12]. In Somalia, these geographic inequities are exacerbated by recurrent environmental shocks, such as droughts, against a backdrop of one of the highest burdens of nutritional deficiency among women of reproductive age [6]. Consequently, the likelihood of a woman being “health poor” is often contingent upon her province of residence and the local socioeconomic climate [11, 12].

Individual‐level factors, such as educational attainment and marital status, intersect with regional variables to influence poverty risk. Education acts as a protective factor, increasing health literacy and the likelihood of formal employment [8, 12]. However, in many patriarchal societies, traditional cultural practices and early marriage limit these opportunities, forcing many women into the informal sector, where they lack social protection [3, 7]. In Somalia, where high fertility rates and low levels of female education persist, understanding how these individual characteristics interact with regional socioeconomic conditions is essential for identifying the most vulnerable populations [13].

Despite the recognized severity of these issues, a granular analysis of the factors driving poverty among Somali women across different regions is still needed. Most existing studies focus on national averages, potentially masking the unique challenges women face in specific administrative areas. This study aims to fill this gap by utilizing national survey data to identify the sociodemographic and regional factors associated with poverty among women of reproductive age in Somalia. By examining these disparities, this study seeks to provide evidence‐based insights to inform targeted public health interventions and poverty alleviation strategies aimed at achieving Sustainable Development Goal (SDG) 1 (No Poverty), SDG 3 (Good Health and Well‐being), and SDG 5 (Gender Equality).

## 2 Methods

### 2.1 Study Design and Data Source

This study employed a cross‐sectional analytical design using data from the 2020 Somalia Demographic and Health Survey (SDHS; officially the Somali Health and Demographic Survey), the first survey of its kind in Somalia [13]. The SDHS is a household survey designed to be nationally representative that adapted standard Demographic and Health Survey (DHS) questionnaires to generate population‐based estimates of health and sociodemographic indicators. The survey used a stratified, multistage cluster sampling design to ensure representativeness across administrative regions and places of residence. Women of reproductive age were interviewed using structured questionnaires that captured household characteristics, demographic profiles, and key maternal and household‐level indicators relevant to living conditions and socioeconomic status.

### 2.2 Sampling Procedure and Study Population

The SDHS 2020 sampling followed a multistage cluster sampling approach. Strata were defined by region and type of place of residence (urban, rural, or nomadic). In urban and rural strata, enumeration areas were selected in two stages with probability proportional to size, and about 30 households were then systematically selected in each selected area; in nomadic strata, temporary nomadic settlements were selected with probability proportional to size, followed by systematic selection of households. Lower Shabelle and Middle Juba, as well as the rural and nomadic areas of Bay, were excluded for security reasons [13]. All ever‐married women aged 12–49 years and never‐married women aged 15–49 years in the selected households were eligible for interview, and the published estimates and public data files are restricted to women aged 15–49 years [13]. Complete‐case analysis was applied; records with missing data across the outcome or explanatory variables were dropped prior to analysis. Because place of delivery is recorded for each birth, the analysis used the SDHS birth recode file, so women with more than one birth could contribute more than one record. Of the 18,561 births with a recorded place of delivery, 5438 (29.3%) were excluded because of missing data, most often on husband's education, household size, or husband's or women's work status. For the present analysis, the study population comprised women aged 15–49 years with a recorded place of delivery and with complete information on the outcome variable (poverty status) and selected explanatory variables. The final analytical sample included 13,123 unweighted observations, with a weighted total of approximately 12,774, distributed across 574 primary sampling units and 44 strata.

### 2.3 Outcome Variable: Poverty Status

The outcome of interest was relative asset‐based poverty among women of reproductive age, operationalized as a binary variable (poor vs. nonpoor). Poverty classification was derived from the SDHS household wealth index (V190), a composite measure constructed using principal component analysis of household assets, housing materials, and access to basic services [13]. Women in the lowest two wealth quintiles (poorest and poorer; quintiles 1 and 2) were classified as poor (coded 1), whereas those in the middle, richer, and richest quintiles (quintiles 3–5) were classified as nonpoor (coded 0). Because wealth quintiles are defined on the household population, this cut‐off identifies the poorest 40% of the population; it was chosen to contrast the most disadvantaged households with all others, consistent with the study's focus on relative asset‐based disadvantage, and it does not represent absolute monetary poverty.

### 2.4 Explanatory Variables

Explanatory variables included individual, household, and geographical characteristics relevant to women's household poverty status and available in the SDHS dataset. Women's age was categorized into seven groups: 15–19, 20–24, 25–29, 30–34, 35–39, 40–44, and 45–49 years. Educational attainment was classified as no education, primary, secondary, or higher. Women's and husbands’/partners’ employment status reflected whether they had worked during the preceding 12 months, categorized as yes or no. Husband/partner schooling was represented by a binary indicator of whether the husband or partner had ever attended school.

Maternal health‐service characteristics included place of delivery, categorized as a health facility or home, and perceived barriers to healthcare access. Healthcare barriers encompassed difficulties obtaining permission, obtaining money for advice or treatment, reaching a health facility because of distance, or attending alone. The composite indicator distinguished no barrier/no problem from at least one barrier/problem. Media exposure was based on the highest reported frequency of radio or television exposure and categorized as weekly or more, less than weekly, or not at all.

Household characteristics included household size, categorized as 4 or fewer, 5–7, or 8–9 members (values above nine are missing in the birth recode file, so these records were excluded), and sex of the household head, classified as male or female. Geographical characteristics comprised residence type—rural, urban, or nomadic—and administrative region. The analysis included 16 regions: Awdal, Woqooyi Galbeed, Togdheer, Sool, Sanaag, Bari, Nugaal, Mudug, Galgaduud, Hiraan, Middle Shabelle, Banadir, Bay, Bakool, Gedo, and Lower Juba. No observations were available for Lower Shabelle or Middle Juba.

### 2.5 Statistical Analysis

Analyses were conducted using Stata version 18.0 (StataCorp LLC, College Station, TX, USA), accounting for the SDHS's complex sampling design, including clustering, stratification, and sampling weights. The survey design was declared using the svyset command, specifying sampling weights, primary sampling units, and strata. Descriptive characteristics were summarized using weighted frequencies and percentages. Overall poverty proportions were estimated with 95% confidence intervals (CIs) using survey‐adjusted procedures (svy: proportion).

For bivariate analysis, poverty status was cross‐tabulated against each explanatory variable, and survey‐adjusted row percentages with 95% CIs were reported. Associations were assessed using design‐based Rao−Scott *F*‐tests from svy: tabulate, with statistical significance defined as a two‐sided *p*‐value <0.05.

Separate survey‐weighted logistic regression models (svy: logistic) estimated crude odds ratios (cORs), while a multivariable model including all explanatory variables simultaneously estimated adjusted odds ratios (aORs). Results were reported with 95% CIs and survey‐adjusted *p*‐values. The design‐adjusted Wald test assessed the joint significance of the predictors (*p* < 0.001). Model fit was assessed with an *F*‐adjusted mean residual goodness‐of‐fit test, which did not indicate lack of fit (*p* > 0.05). Multicollinearity was examined using an auxiliary linear regression with numerically coded predictors (mean variance inflation factor [VIF] = 1.10; maximum = 1.28); these values describe the auxiliary specification and do not assess collinearity among the categorical terms in the final model. Generalized variance inflation factors (GVIFs) for the categorical terms in the final model were also low (maximum degrees‐of‐freedom‐adjusted GVIF = 1.18).

### 2.6 Ethical Considerations

The SDHS 2020 protocols were approved by the relevant national and institutional ethics review bodies, and informed consent was obtained from all participants before data collection. This study used deidentified publicly available SDHS data obtained from the Somali National Bureau of Statistics through the Somali National Data Archive. As this analysis involved the secondary use of anonymized survey data, it did not require additional participant consent and posed minimal risk to respondents.

## 3 Results

### 3.1 Poverty Prevalence

The overall prevalence of poverty within the analytical sample (unweighted *n* = 13,123; weighted *N* ≈ 12,774), accounting for the survey design, was estimated to be 48.5% (95% CI: 42.4%–54.6%). Consequently, 51.5% (95% CI: 45.4%–57.6%) of the women in this sample were classified as nonpoor.

### 3.2 Sociodemographic Characteristics of the Study Population

The sociodemographic profile highlights significant structural vulnerabilities in the study population (Table 1). The age distribution was relatively young, with the largest proportion of women falling into the 25–29 years age group (31.19%). A vast majority (85.62%) of the women lacked formal education, and only a negligible proportion (0.83%) reported working in the 12 months before the survey. In contrast, 39.94% of husbands/partners were reported to work during the same period. High rates of home delivery were prevalent, with 81.60% of women delivering at home rather than in a health facility. Regarding healthcare access, 76.77% of respondents reported facing barriers/problems, whereas 23.23% reported no barriers. Media exposure was critically low, with 87.16% of respondents reporting no exposure to radio or television. Regarding residence, the population was distributed across urban (61.27%), rural (25.97%), and nomadic settings (12.77%).

**TABLE 1:** Sociodemographic characteristics of women in the analytical sample.

Characteristic	Category	Frequency (weighted *N*)	Percentage (%)
Age group (years)	15–19	561	4.39
	20–24	2679	20.97
	25–29	3984	31.19
	30–34	2685	21.02
	35–39	1986	15.55
	40–44	702	5.50
	45–49	177	1.38
Educational level	No education	10,937	85.62
	Primary	1457	11.41
	Secondary	284	2.23
	Higher	96	0.75
Women worked in the preceding 12 months	Yes	106	0.83
	No	12,669	99.17
Husband worked	Yes	5102	39.94
	No	7673	60.06
Husband education	No	1919	15.02
	Yes	10,856	84.98
Place of delivery	Health facility	2351	18.40
	Home	10,424	81.60
Healthcare access	No barrier/no problem	2968	23.23
	Barrier/problem	9807	76.77
Media exposure	Weekly or more	1186	9.29
	Less than weekly	454	3.55
	Not at all	11,134	87.16
Household size	≤4	4248	33.26
	5–7	5998	46.96
	8‐9	2528	19.79
Sex of household head	Male	8477	66.36
	Female	4298	33.64
Residence	Rural	3317	25.97
	Urban	7826	61.27
	Nomadic	1631	12.77

*Note:* Frequencies and percentages are survey‐weighted; frequencies are rounded to whole numbers. Totals may vary slightly due to rounding.

### 3.3 Regional Disparities in Poverty

The analysis revealed regional disparities in the prevalence of poverty among women (Figure 1). Poverty rates varied substantially across administrative regions (*p* = 0.011). As illustrated in the figure, the highest poverty concentrations were observed in Gedo (73.8%), Woqooyi Galbeed (69.9%), Nugaal (68.9%), and Bari (63.3%). Conversely, the lowest poverty rates were recorded in Bay (28.2%; urban areas only), Sanaag (30.4%), and Lower Juba (31.4%). The regions of Togdheer (41.9%) and Banadir (41.9%) displayed moderate poverty levels, relative to the national average.

**FIGURE 1:**
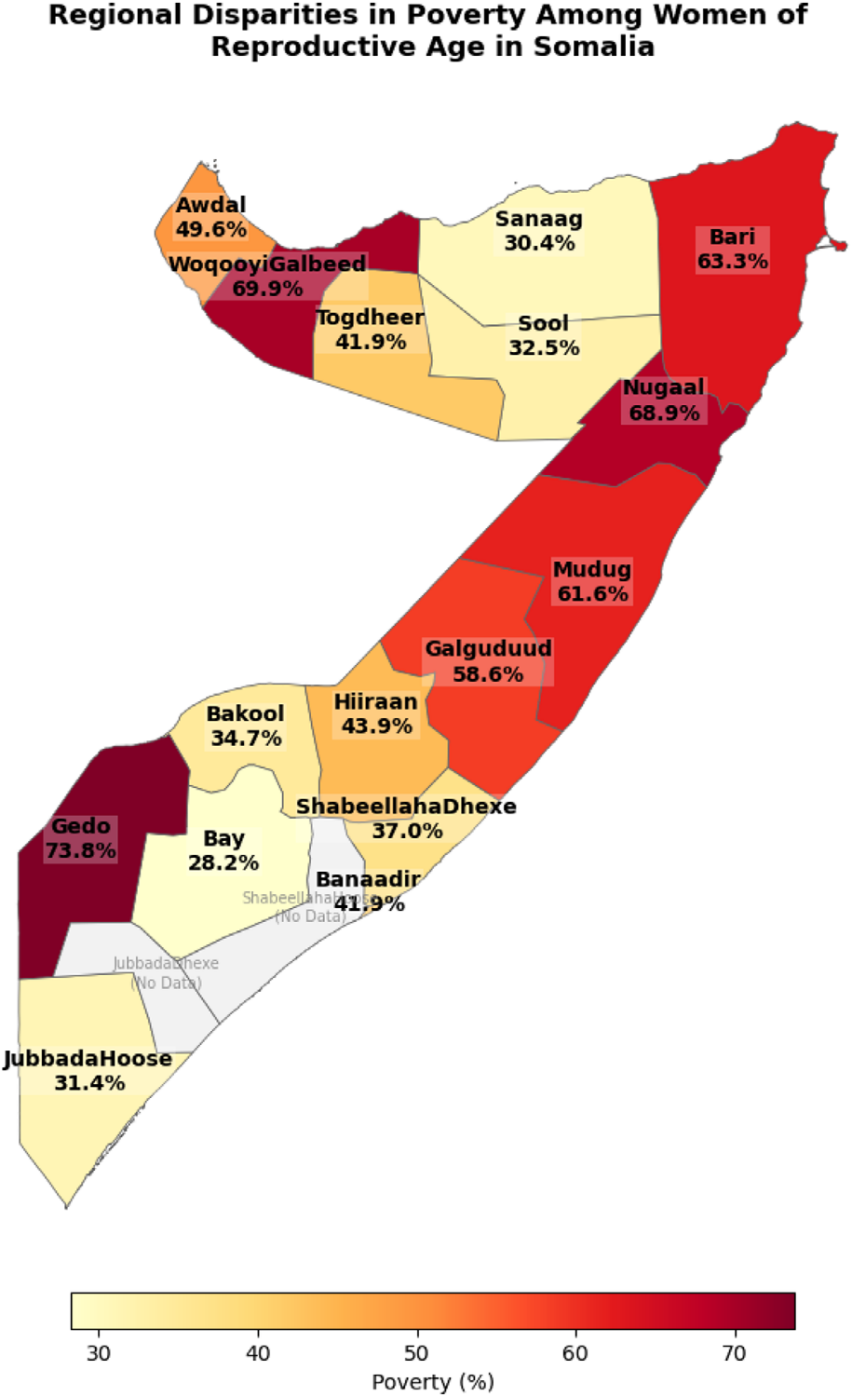
Regional disparities of poverty prevalence among women of reproductive age in Somalia.

### 3.4 Bivariate Analysis: Factors Associated With Poverty

Table 2 presents the survey‐adjusted distribution of poverty status across the predictor variable categories. Row percentages with 95% CIs and design‐based *F*‐statistics assessing the association are shown. The survey‐adjusted bivariate analysis indicated noteworthy associations between poverty and the place of delivery, healthcare access, maternal education, media exposure, and husbands’ characteristics. Notably, strong gradients were observed in education and media exposure. Women with no education were significantly more likely to be poor (54.6%) than those with higher education (2.4%). Similarly, women with no media exposure had a poverty prevalence of 53.8%, compared with 11.3% among those with weekly exposure. Women who delivered at home had a substantially higher poverty rate (55.9%) than those who delivered in health facilities (15.6%). Factors that were not significantly associated with poverty in the bivariate analysis included age group, women's working status, household size, sex of household head, and type of residence.

**TABLE 2:** Bivariate associations between predictor variables and poverty status.

Characteristic	Category	Nonpoor % [95% CI]	Poor % [95% CI]	p‐value
**Overall prevalence**	**Total**	**51.5 [45.4, 57.6]**	**48.5 [42.4, 54.6]**	
**Age group (years)**	15–19	50.2 [40.9, 59.5]	49.8 [40.5, 59.1]	0.052
	20–24	51.9 [45.3, 58.5]	48.1 [41.5, 54.7]	
	25–29	53.2 [45.7, 60.6]	46.8 [39.4, 54.3]	
	30–34	53.3 [45.6, 60.8]	46.7 [39.2, 54.4]	
	35–39	50.5 [43.8, 57.2]	49.5 [42.8, 56.2]	
	40–44	40.5 [32.9, 48.5]	59.5 [51.5, 67.1]	
	45–49	40.5 [29.9, 52.1]	59.5 [47.9, 70.1]	
**Educational level**	No education	45.4 [39.4, 51.6]	54.6 [48.4, 60.6]	<0.001
	Primary	85.2 [80.4, 89.0]	14.8 [11.0, 19.6]	
	Secondary	97.3 [90.4, 99.3]	2.7 [0.7, 9.6]	
	Higher	97.6 [84.2, 99.7]	2.4 [0.3, 15.8]	
**Place of delivery**	Health facility	84.4 [80.2, 87.8]	15.6 [12.2, 19.8]	<0.001
	Home	44.1 [37.6, 50.9]	55.9 [49.1, 62.4]	
**Healthcare access**	No barrier/no problem	68.9 [62.3, 74.8]	31.1 [25.2, 37.7]	<0.001
	Barrier/problem	46.3 [40.1, 52.5]	53.7 [47.5, 59.9]	
**Husband worked**	Yes	75.5 [69.4, 80.7]	24.5 [19.3, 30.6]	<0.001
	No	35.6 [29.2, 42.5]	64.4 [57.5, 70.8]	
**Husband education**	No	87.8 [84.2, 90.7]	12.2 [9.3, 15.8]	<0.001
	Yes	45.1 [38.9, 51.5]	54.9 [48.5, 61.1]	
**Media exposure**	Weekly or more	88.7 [82.8, 92.7]	11.3 [7.3, 17.2]	<0.001
	Less than weekly	84.4 [73.9, 91.2]	15.6 [8.8, 26.1]	
	Not at all	46.2 [39.7, 52.9]	53.8 [47.1, 60.3]	
**Women worked in the preceding 12 months**	Yes	67.2 [46.2, 83.0]	32.8 [17.0, 53.8]	0.120
	No	51.4 [45.2, 57.5]	48.6 [42.5, 54.8]	
**Residence**	Rural	50.3 [41.9, 58.8]	49.7 [41.2, 58.1]	0.785
	Urban	51.2 [42.1, 60.2]	48.8 [39.8, 57.9]	
	Nomadic	55.7 [44.7, 66.1]	44.3 [33.9, 55.3]	
**Household size**	≤4	50.6 [44.2, 57.0]	49.4 [43.0, 55.8]	0.569
	5–7	51.2 [44.4, 58.1]	48.8 [41.9, 55.6]	
	8–9	53.7 [46.4, 60.8]	46.3 [39.2, 53.6]	
**Sex of household head**	Male	52.7 [46.4, 58.9]	47.3 [41.1, 53.6]	0.118
	Female	49.2 [42.5, 56.0]	50.8 [44.0, 57.5]	
**Region**	Awdal	50.4 [36.7, 64.1]	49.6 [35.9, 63.3]	0.011
	Woqooyi Galbeed	30.1 [16.9, 47.7]	69.9 [52.3, 83.1]	
	Togdheer	58.1 [42.4, 72.4]	41.9 [27.6, 57.6]	
	Sool	67.5 [55.8, 77.3]	32.5 [22.7, 44.2]	
	Sanaag	69.6 [60.2, 77.7]	30.4 [22.3, 39.8]	
	Bari	36.7 [23.6, 52.1]	63.3 [47.9, 76.4]	
	Nugaal	31.1 [18.0, 48.3]	68.9 [51.7, 82.0]	
	Mudug	38.4 [21.2, 59.0]	61.6 [41.0, 78.8]	
	Galgaduud	41.4 [21.5, 64.6]	58.6 [35.4, 78.5]	
	Hiraan	56.1 [40.6, 70.4]	43.9 [29.6, 59.4]	
	Middle Shabelle	63.0 [43.3, 79.1]	37.0 [20.9, 56.7]	
	Banadir	58.1 [40.9, 73.5]	41.9 [26.5, 59.1]	
	Bay	71.8 [37.1, 91.6]	28.2 [8.4, 62.9]	
	Bakool	65.3 [48.1, 79.2]	34.7 [20.8, 51.9]	
	Gedo	26.2 [11.8, 48.6]	73.8 [51.4, 88.2]	
	Lower Juba	68.6 [42.0, 86.9]	31.4 [13.1, 58.0]	

*Note:* A design‐based Rao−Scott *F*‐statistic was used to assess associations.

Abbreviation: CI, confidence interval.

### 3.5 Multivariable Analysis: Independent Determinants of Poverty

A multivariable survey logistic regression model was employed to identify the independent predictors of poverty, adjusting for potential confounders. Table 3 presents both cORs and aORs, along with their 95% CIs, and *p*‐values. Higher levels of maternal education were significantly associated with lower odds of poverty. Women with primary (aOR = 0.31, 95% CI: 0.23–0.44), secondary (aOR = 0.08, 95% CI: 0.02–0.36), and higher (aOR = 0.09, 95% CI: 0.01–0.92) education had substantially lower odds of being poor than women with no education.

**TABLE 3:** Crude and adjusted survey‐weighted logistic regression results for factors associated with poverty among women of reproductive age.

Variable	Unadjusted cOR	95% CI	*p*‐value	Adjusted aOR	95% CI	*p*‐value
**Age group (Ref: 15–19)**						
20–24	0.93	[0.66, 1.33]	0.707	1.18	[0.77, 1.81]	0.437
25–29	0.89	[0.63, 1.26]	0.499	0.92	[0.62, 1.36]	0.664
30–34	0.89	[0.62, 1.27]	0.512	0.84	[0.57, 1.25]	0.396
35–39	0.99	[0.67, 1.45]	0.957	0.88	[0.55, 1.39]	0.574
40–44	1.49	[0.99, 2.23]	0.055	1.10	[0.71, 1.70]	0.681
45–49	1.48	[0.86, 2.56]	0.158	1.20	[0.64, 2.23]	0.569
**Place of delivery (Ref: Health facility)**						
Home	6.85	[5.13, 9.14]	<0.001	3.90	[2.94, 5.18]	<0.001
**Healthcare access (Ref: No barrier/no problem)**						
Barrier/problem	2.57	[2.09, 3.16]	<0.001	2.36	[1.84, 3.02]	<0.001
**Educational level (Ref: No education)**						
Primary	0.14	[0.11, 0.19]	<0.001	0.31	[0.23, 0.44]	<0.001
Secondary	0.02	[0.01, 0.09]	<0.001	0.08	[0.02, 0.36]	0.001
Higher	0.02	[0.00, 0.16]	<0.001	0.09	[0.01, 0.92]	0.042
**Women worked (Ref: Yes)**						
No	1.94	[0.83, 4.52]	0.127	0.88	[0.35, 2.20]	0.789
**Media exposure (Ref: Weekly or more)**						
Less than weekly	1.44	[0.69, 3.02]	0.330	1.46	[0.63, 3.39]	0.381
Not at all	9.11	[5.82, 14.26]	<0.001	4.47	[2.92, 6.83]	<0.001
**Husband worked (Ref: Yes)**						
No	5.57	[4.16, 7.48]	<0.001	4.18	[3.28, 5.31]	<0.001
**Husband education (Ref: No)**						
Yes	8.75	[6.48, 11.81]	<0.001	3.69	[2.67, 5.11]	<0.001
**Household size (Ref: ≤4)**						
5–7	0.98	[0.81, 1.17]	0.793	1.12	[0.91, 1.37]	0.297
8–9	0.88	[0.69, 1.13]	0.315	0.95	[0.74, 1.23]	0.718
**Sex of household head (Ref: Male)**						
Female	1.15	[0.97, 1.37]	0.118	1.09	[0.92, 1.29]	0.297
**Region (Ref: Awdal)**						
Woqooyi Galbeed	2.36	[0.92, 6.05]	0.073	1.35	[0.50, 3.68]	0.556
Togdheer	0.73	[0.31, 1.71]	0.472	0.48	[0.19, 1.18]	0.110
Sool	0.49	[0.23, 1.04]	0.063	0.31	[0.13, 0.75]	0.009
Sanaag	0.44	[0.22, 0.89]	0.023	0.29	[0.13, 0.69]	0.005
Bari	1.75	[0.75, 4.08]	0.191	0.67	[0.25, 1.77]	0.419
Nugaal	2.25	[0.90, 5.64]	0.083	0.76	[0.27, 2.16]	0.613
Mudug	1.64	[0.59, 4.50]	0.339	0.72	[0.22, 2.36]	0.585
Galgaduud	1.44	[0.48, 4.34]	0.515	0.49	[0.13, 1.90]	0.300
Hiraan	0.80	[0.34, 1.85]	0.597	0.37	[0.13, 1.04]	0.060
Middle Shabelle	0.60	[0.22, 1.60]	0.304	0.39	[0.13, 1.16]	0.092
Banadir	0.73	[0.30, 1.79]	0.496	0.23	[0.08, 0.67]	0.007
Bay	0.40	[0.08, 1.92]	0.251	0.12	[0.02, 0.66]	0.015
Bakool	0.54	[0.22, 1.34]	0.183	0.28	[0.10, 0.80]	0.017
Gedo	2.87	[0.93, 8.88]	0.068	1.12	[0.33, 3.79]	0.856
Lower Juba	0.46	[0.13, 1.61]	0.227	0.23	[0.05, 0.97]	0.046
**Residence (Ref: Rural)**						
Urban	0.97	[0.59, 1.60]	0.897	1.25	[0.71, 2.23]	0.440
Nomadic	0.81	[0.46, 1.41]	0.450	0.99	[0.55, 1.77]	0.970

Abbreviations: aOR, adjusted odds ratio; cOR, crude odds ratio; 95% CI, 95% confidence interval; *p*‐value, probability value; Ref, reference category.

Conversely, lack of media exposure and the husband's unemployment were identified as strong risk factors. Women with no media exposure had over four times the odds of poverty (aOR = 4.47, 95% CI: 2.92–6.83) relative to those with weekly exposure. Similarly, women whose husbands did not work in the last 12 months had significantly higher odds of poverty (aOR = 4.18, 95% CI: 3.28–5.31). Place of delivery remained a robust predictor; women delivering at home had nearly four times the odds of poverty (aOR = 3.90, 95% CI: 2.94–5.18) compared to those delivering in a health facility. Furthermore, women reporting barriers/problems accessing healthcare had more than twice the odds of poverty (aOR = 2.36, 95% CI: 1.84–3.02) compared to those reporting no barrier/no problem.

Regional variables showed significant independent associations after adjustment for confounding factors. Compared to the reference region of Awdal, women in regions such as Sool (aOR = 0.31), Sanaag (aOR = 0.29), Banadir (aOR = 0.23), Bay (aOR = 0.12), Bakool (aOR = 0.28), and Lower Juba (aOR = 0.23) demonstrated significantly lower odds of poverty. Demographic factors such as maternal age, respondent's working status, household size, sex of the household head, and type of residence were not significantly associated with poverty in the adjusted model.

## 4 Discussion

The primary objective of this study was to identify regional disparities and socioeconomic determinants of poverty among Somali women of reproductive age using national survey data from the 2020 SDHS. We found an overall poverty prevalence of 48.5% (95% CI: 42.4%–54.6%), characterized by profound, subnational variation. Multivariable analysis identified maternal education, media exposure, husbands’ employment status, place of delivery, and barriers to healthcare access as significant independent predictors of poverty. These findings offer a unique baseline for understanding how institutional fragility and gendered social structures intersect with economic vulnerability in a post‐conflict setting.

Because poverty was defined relative to the national wealth distribution, about 40% of the household population falls into the poor group by construction; the prevalence of 48.5%, therefore, describes the position of the analytical sample within this distribution rather than an absolute poverty rate and is not directly comparable with monetary poverty rates from other countries. For context, national consumption‐based estimates show that 54% of Somalis lived below the national poverty line in 2022, including more than three‐quarters of the nomadic population [14]. Fragility and gender inequality also shape women's welfare: across Muslim‐majority countries, weak state governance, conflict, and limited empowerment of women and girls have been strongly associated with child and newborn mortality [15], and in sub‐Saharan Africa, women with no education are much less likely to take part in reproductive health decisions [16]. Furthermore, the wide regional variation observed in Somalia mirrors the marked subnational inequalities in maternal health service coverage documented in other African settings, such as Angola [17].

Regarding maternal education, our study found that women with secondary education had 92% lower odds of poverty (aOR = 0.08), and those with higher education had 91% lower odds (aOR = 0.09) compared to those with no formal education. This may reflect the role of education in enhancing women's labor market agency, financial literacy, and bargaining power within households. In the Somali context, even primary schooling was associated with substantially lower odds of poverty (aOR = 0.31), possibly by facilitating access to informal and formal economic networks vital for survival in fragile economies. These findings are consistent with multicountry evidence linking women's empowerment to child and newborn survival in Muslim‐majority countries and women's education to greater participation in reproductive health decisions in sub‐Saharan Africa [15, 16]. In Angola, for example, antenatal care coverage was lower among less educated and poorer women [17].

In terms of information access, women with no media exposure demonstrated 4.47 times higher odds of poverty (aOR = 4.47, 95% CI: 2.92–6.83) than those with weekly exposure. This may indicate an “information deficit,” in which limited access to radio, television, or digital platforms makes it harder for women to identify market opportunities, employment openings, or humanitarian resources. Part of this association may also be mechanical, because household ownership of a radio or television contributes to the wealth index. Media access may also serve as a conduit for shifting gender norms and empowering women to challenge economic marginalization.

Regarding household labor dynamics, women whose husbands were unemployed had 4.18 times higher odds of poverty (aOR = 4.18, 95% CI: 3.28–5.31) than those with employed husbands. This finding highlights the extreme economic dependence of Somali women, given that only 0.83% of women in our sample reported working in the preceding 12 months. In a patriarchal, fragile state where security risks and cultural norms restrict women's mobility, male employment may remain the main protection against household poverty. Similarly, a recent Nigerian study identified the household head's employment status as a significant predictor of vulnerability to poverty [18].

With respect to maternal health utilization, women who delivered at home had 3.90 times higher odds of poverty (aOR = 3.90, 95% CI: 2.94–5.18) compared to those who delivered in health facilities. This may reflect a “health‐poverty trap,” in which limited financial resources lead women to deliver at home, which may in turn increase the risk of catastrophic health expenditures from complications, further entrenching the household in poverty. Previous studies have identified distance and travel time as important barriers to facility delivery [19, 20], and our findings suggest that household economic status is also closely linked to place of delivery in Somalia. These results are consistent with reviews identifying household wealth as one of the factors most consistently associated with facility delivery [20, 21].

Regarding healthcare equity, women reporting barriers/problems accessing healthcare had 2.36 times higher odds of poverty (aOR = 2.36, 95% CI: 1.84–3.02) compared to those reporting no barrier/no problem. Systemic barriers, such as high costs, lack of transport, and the absence of female providers, may disproportionately affect poor women, creating a possible bidirectional relationship in which poverty prevents care‐seeking and lack of care prevents economic recovery. This finding is consistent with persistent socioeconomic inequalities in access to maternal health services in South Africa [22] and with evidence from Ethiopia that antenatal care use strongly increases institutional delivery [23]. Omar et al. similarly highlighted socioeconomic and geographic barriers to optimal antenatal care use in South and Central Somalia [24].

Finally, our analysis of regional disparities revealed that poverty prevalence ranged from 28.2% in Bay to 73.8% in Gedo, with significant aORs showing lower odds of poverty in Bay (aOR = 0.12, 95% CI: 0.02–0.66) and Banadir (aOR = 0.23, 95% CI: 0.08–0.67) than in Awdal. These disparities may reflect differences in the localized intensity of conflict, the quality of regional governance, and access to infrastructure. For instance, Banadir is entirely urban and may benefit from better access to services, whereas the estimate for Bay reflects only its urban areas (rural and nomadic Bay were not surveyed [13]) and is imprecise (95% CI: 8.4%–62.9%). National consumption‐based estimates also show marked geographic differences in poverty within Somalia [14].

These findings suggest a need for integrated, multisectoral interventions tailored to the Somali context, although they should be interpreted cautiously because the data are cross‐sectional and place of delivery and barriers to care may be consequences as well as causes of poverty. Given the 92% lower odds of poverty associated with secondary education, policymakers could consider eliminating school fees and providing conditional cash transfers to keep girls in school. Simultaneously, supporting dual‐income households through gender‐sensitive microfinance and vocational training may be important, given the 4.18 times higher odds of poverty associated with male unemployment. Expanding radio and digital infrastructure may be a high‐impact, low‐cost priority; mobile‐based information platforms could help bridge the media gap, given the 4.47 times higher odds of poverty among women without media exposure. Furthermore, eliminating user fees for maternal health services and deploying mobile clinics to high‐poverty regions such as Gedo may be important, given the 3.90 times higher odds of poverty associated with home delivery. Finally, interventions may be more effective if they are geographically targeted. High‐poverty regions may require conflict‐sensitive humanitarian aid and infrastructure investment, while urban areas, such as Banadir, could focus on formalizing women's economic participation and digital literacy.

This study has several notable strengths, primarily the use of national survey data from the 2020 Somalia Demographic and Health Survey (SDHS), the first survey of its kind in Somalia, which provides a valuable baseline on women's health and household circumstances. The large sample size provided adequate statistical power to detect meaningful associations, and the use of survey‐weighted multivariable regression accounted for the complex sampling design.

Despite these strengths, several limitations must be considered. First, the cross‐sectional nature of the data restricts the ability to establish temporal relationships or causal inferences between predictors and poverty outcomes. Second, the measurement constraints include potential recall and social desirability biases inherent in self‐reported data. Third, the binary poverty classification (poor vs. nonpoor) may simplify the multidimensional nature of deprivation, which encompasses health, education, nutrition, and social capital. Dichotomizing the wealth quintiles also obscures differences within the poor and nonpoor groups and does not capture the full wealth gradient. Fourth, the SDHS wealth index is an asset‐based relative measure; although the household questionnaire recorded livestock ownership and herd sizes, the index also draws heavily on housing materials (e.g., concrete flooring, brick walls) and fixed municipal utilities (e.g., piped drinking water, electricity). In the Somali context, where nomadic residents made up nearly 13% of our weighted analytical sample, pastoralist household wealth is primarily held in mobile, liquid livestock assets (such as camels, goats, sheep, and cattle) and informal diaspora remittances. Consequently, asset‐based indices may underestimate the actual economic capital of pastoralist households, potentially misclassifying some nomadic pastoralists as asset‐poor. In the SDHS 2020 report, about 95% of the nomadic population fell in the two lowest wealth quintiles [13], so this possible misclassification warrants caution when interpreting differences across residence groups. Fifth, because place of delivery was required, the sample was restricted to women with a recorded birth, so the findings may not apply to all women of reproductive age. Finally, the analysis did not include critical external factors such as active conflict exposure, displacement status, or clan‐based safety nets, all of which strongly influence household resilience in Somalia. Collectively, these limitations underscore the need for future longitudinal and mixed‐methods research to better capture the structural, cultural, and contextual drivers of economic vulnerability and resilience in fragile settings.

## 5 Conclusion

Marked regional inequities in poverty were observed among women of reproductive age in Somalia, with deprivation concentrated in specific administrative areas. Educational attainment was strongly associated with lower odds of poverty, suggesting that even modest schooling may be linked to reduced economic vulnerability. Limited access to information, reflected by low media exposure, aligns with greater poverty, suggesting that informational isolation may restrict awareness of opportunities, services, and support systems. Household labor dynamics also mattered, with women in households where partners lacked work facing higher odds of poverty, possibly reflecting the structural dependence created by constrained female employment opportunities. Maternal health service use and perceived access to care were closely linked to poverty, possibly reflecting a reinforcing cycle in which financial hardship limits service utilization and weakens resilience to health‐related shocks. These patterns highlight how gendered disadvantage in fragile settings may be produced through the combined effects of place, education, information, livelihoods, and access to essential services. Therefore, addressing poverty among women may require strategies that extend beyond short‐term assistance and tackle the upstream social and system determinants that shape women's agency and household security. Priority actions include keeping girls in school through fee removal and targeted support, expanding women's access to information through community and mobile communication channels, and strengthening household livelihoods through employment and skills pathways that are feasible in insecure and resource‐limited contexts. Reducing financial and practical barriers to maternal healthcare, including community outreach and service availability in high‐need areas, may help disrupt the cycle of health poverty. Future work should incorporate longitudinal designs and contextual measures, such as displacement and insecurity, to clarify pathways and guide geographically tailored interventions that can accelerate progress toward equitable development outcomes.

## Author Contributions

MMA conceptualized the study. The study design was developed collaboratively by ASA, MMA, MAO, and YSAH. Data acquisition and preparation were performed by YSAH and MMA. MAO analyzed and interpreted the data. The initial draft was composed by ASA, MMA, and YSAH. All authors contributed to the writing, reviewing, and editing of the subsequent versions of the manuscript.

## Funding

The authors have not received any funding for this study.

## Ethics Approval and Consent to Participate

This study is a secondary analysis of the publicly available and fully anonymized 2020 Somalia Demographic and Health Survey (SDHS) dataset. The original survey protocol was reviewed and approved by the relevant institutional review board in Somalia (Ministry of Health and Human Services and the Somali National Bureau of Statistics), and all participants provided informed consent prior to data collection. As our study used no identifiable private information, it was exempt from further institutional ethical review.

## Consent for Publication

This study analyzes secondary data and does not involve individually identifiable information.

## Conflicts of Interest

The authors declare no competing interests.

## Data Availability

The data that support the findings of this study are available from the 2020 Somalia Health and Demographic Survey (SHDS), referred to as the SDHS in this article. These data are publicly available upon registration from the Somali National Data Archive (SoNADA) and can be accessed at https://microdata.nbs.gov.so/index.php/catalog/50. The dataset reference number is SOM‐SNBS‐SHDS‐2020‐v01.
